# DomeVR: Immersive virtual reality for primates and rodents

**DOI:** 10.1371/journal.pone.0308848

**Published:** 2025-01-16

**Authors:** Katharine A. Shapcott, Marvin Weigand, Mina Glukhova, Martha N. Havenith, Marieke L. Schölvinck

**Affiliations:** Ernst Strüngmann Institute (ESI) for Neuroscience in Cooperation with the Max Planck Society, Frankfurt-am-Main, Germany; Galgotias University, INDIA

## Abstract

Immersive virtual reality (VR) environments are a powerful tool to explore cognitive processes ranging from memory and navigation to visual processing and decision making—and to do so in a naturalistic yet controlled setting. As such, they have been employed across different species, and by a diverse range of research groups. Unfortunately, designing and implementing behavioral tasks in such environments often proves complicated. To tackle this challenge, we created DomeVR, an immersive VR environment built using Unreal Engine 4 (UE4). UE4 is a powerful game engine supporting photo-realistic graphics and containing a visual scripting language designed for use by non-programmers. As a result, virtual environments are easily created using drag-and-drop elements. DomeVR aims to make these features accessible to neuroscience experiments. This includes a logging and synchronization system to solve timing uncertainties inherent in UE4; an interactive GUI for scientists to observe subjects during experiments and adjust task parameters on the fly, and a dome projection system for full task immersion in non-human subjects. These key features are modular and can easily be added individually into other UE4 projects. Finally, we present proof-of-principle data highlighting the functionality of DomeVR in three different species: human, macaque and mouse.

## Introduction

In recent years, it has become abundantly clear that the study of brain activity will benefit enormously from immersive naturalistic tasks [1–6]. Traditionally, neuroscience has taken a reductionist approach: rigorous experiments with simplified stimuli were designed to dissect out single cognitive processes, such as attention [7] or memory [8], and their neuronal underpinnings. Even though this approach has been immensely successful in explaining brain activity acquired during such experiments, it is hardly reflective of the highly dynamic and varied environment that the brain encounters in everyday life. Indeed, the use of more naturalistic visual input has forced us to revise fundamental notions about the brain, such as the receptive field [9–12]. However, truly naturalistic settings (i.e., animals freely roaming through a complex environment) limit experimental control. Precise tracking of eye movements and the exact timings of events to relate to neural activity are possible, but experimentally extremely challenging in these circumstances [13, 14]. To circumvent this, multiple labs have harnessed virtual reality (VR) [15–18] to create immersive naturalistic tasks while still allowing for precise experimental control. Such virtual reality environments are highly effective at creating a feeling of presence in the virtual world as long as certain minimal conditions are met [19], and are therefore a close proxy to truly naturalistic settings. However, VR programs typically either require a high level of coding proficiency (VR software used in primates) or feature quite rudimentary VR environments (VR paradigms applied in rodents). We therefore set out to develop a VR toolbox that is easy to use by non-programmers while also generating naturalistic VR environments with the high timing precision required to be compatible with neuronal recordings. What’s more, we designed our toolbox to flexibly interface with different types of movement sensors such as joysticks and track balls in order to support experiments across various model species.

We chose to create our VR toolbox using Unreal Engine 4 (UE4) [www.unrealengine.com]. UE4 is a free state-of-the-art game engine with open source code, geared towards designing realistic VR environments. Importantly, unlike other game engines such as Unity [www.unity.com], UE4 contains a mature visual scripting language that makes coding for non-programmers more accessible. Like all game engines, UE4 lacks features necessary for performing controlled behavioral experiments, such as precise timing control. To implement these features in UE4, we developed DomeVR. DomeVR is a modular toolbox containing features necessary to create immersive behavioral tasks for different species, control them during execution, and analyze the resulting behavioral data. Specifically, DomeVR has the following features:

1. **Objects that can be placed in the virtual environment and serve as a part of tasks (e.g., a stimulus)**.
2. **Visual scripting features to control task flow via state machines with blocks and trials**.
3. **Timing synchronization to ensure data can be aligned to neural activity**.
4. **A logging system to automatically record relevant information needed for offline analysis**.
5. **Logging analysis in python**.
6. **A dome projection suitable for immersive human and non-human VR experiments**.
7. **Real-time inputs and outputs (e.g., eye tracking and “event markers” (unique numbers to synchronize events))**.
8. **An experiment GUI to control task parameters online and display behavioral performance**.

The dome projection, eyetracking, event markers and the log with timing synchronization and analysis are available as independent modules that can be added into any UE4 project. You can find the code for the full DomeVR Toolbox and the extensible UE4 plugins at www.github.com/zero-noise-lab.

In the following sections, we briefly describe UE4, then detail the features of DomeVR individually. Finally, we illustrate their application in a two-alternative forced choice task performed by mice, monkeys and humans in a dome environment suitable for all three species (see Results).

## Materials and methods

### Unreal Engine 4

**Fig 1:**
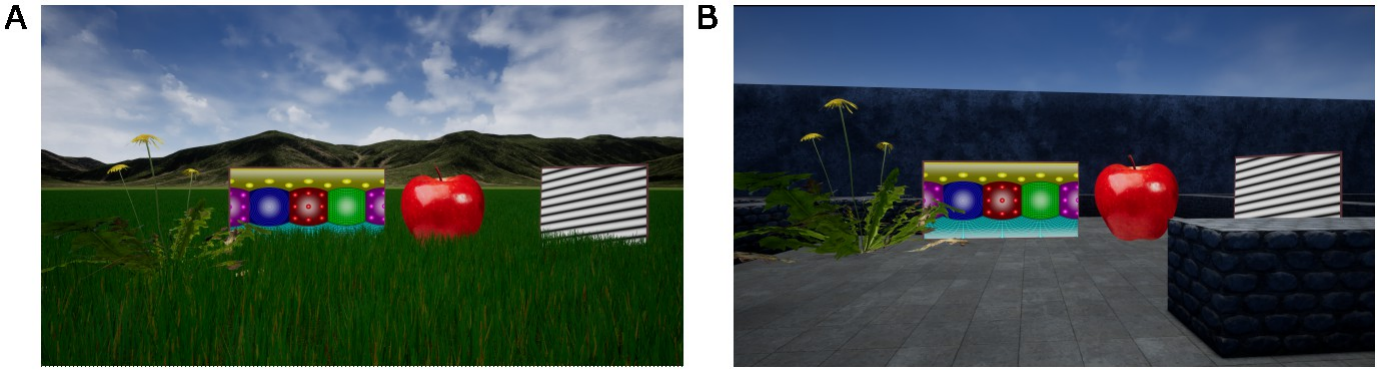
Example levels made with Unreal. (A) Examples of four DomeVR Stimulus types added to a foliage filled Level. From left to right: MeshStimulus, MovieStimulus, ImageStimulus, GratingStimulus. The foliage was generated with UE4 landscape grass types. (B) As A but for a level with a maze.

To create DomeVR, we made use of two crucial features of UE4: its excellent graphics to create immersive VR environments, and its easy-to-use scripting language to efficiently create experimental task flows. To create a VR environment, called a “Level”, an extensive GUI is available in UE4. We created multiple Levels using this GUI, and show two examples in Fig 1, one with foliage and a complex background, another with walls creating a maze. UE4 also allows users to make very large and realistic Levels. Our experiments required immersive VR environments, and we therefore created an eight km long grassy field filled with foliage by using UE4’s landscape grass types, which provided the information for dynamic spawning of foliage. If the spawned foliage was too far away, it was either shown at a lower level of detail or automatically deleted, thus preserving performance at run-time even in large levels.

These large Levels can be filled with many possible objects; walls, forests, etc. We used the drag-and-drop feature of the UE4 GUI to place objects into the Levels, for example the “Player” (the game character that is controlled by the experimental subject). The Player can move through the created Level via an input device (e.g., keyboard or joystick) and can collide with other objects in the Level. Populating VR environments with different objects by simply dragging and dropping them into a Level allows for straightforward and intuitive implementation of behavioral task designs.

To create our experimental task, objects in a Level have to interact. For this, custom code must be added to the objects; for example, an object could appear in front of the Player after a variable delay. Even though UE4 is written in C++, it provides a visual scripting language called Blueprint, which can be used inside the UE4 GUI and enables non-programmers to add code to objects easily by dragging and dropping. Blueprint code encompasses all the elements found in a usual text based programming language including variable declarations, functions, control structures such as for-loops, delegates and class definitions. Although the code is interpreted, it is compiled into fast byte code, which offers a performance that is appropriate for real-time computer graphics in most situations. Interpreted code does not require a lengthy compilation procedure and thus enables rapid testing of the written code. We made use of Blueprints extensively and only used C++ for performance-critical parts or low-level code that would not be possible to code in Blueprints.

The DomeVR functionalities described below (stimuli, state machines, control flow, timing control, and behavioral logging) allow one to use DomeVR for a VR experiment in any standard experimental setup. For example, UE4 can display tasks on a computer screen or using VR goggles.

### Code accessibility

The code/software described in the paper is freely available online at www.github.com/zero-noise-lab.

### Stimuli

In order to fill Levels with objects or images in a controlled manner, we created several classes of stimuli in DomeVR. The four most common examples (ImageStimulus, MovieStimulus, GratingStimulus, and MeshStimulus) are shown in Fig 1). We created all types of stimuli from a stimulus base class, which enables any number of classes to inherit its properties. For the stimulus base class, these properties include parameters like scale (which sets the size of the stimulus), height (which sets the height of the stimulus from the ground), and hide (which determines its visibility). The parameters can be easily set by the user using Blueprints, and with some coding experience additional stimulus classes could be created. All our DomeVR Blueprint or C++ code classes are derived from UE4 base classes, all of which (as well as their relationships to each other) are outlined in S1 Fig. Once a stimulus class is created, any number of stimuli, all with individually set parameters, can be added to a Level. To define a particular stimulus and its parameters, we created one master struct called StimulusSettings. StimulusSettings defines the basic parameters common to all stimuli and defines which of the stimulus types it is. This has the advantage that the experimenter can switch between stimulus types in the same state by simply changing the stimulus type parameter. Each stimulus type has its own struct with specific stimulus parameters which can be applied to the StimulusSettings struct. These parameters are saved in the log automatically as outlined in Behavioral logging.

As one can imagine, each stimulus class works with a different set of parameters and functions. For example, the MeshStimulus class is for displaying 3D objects on the fly in UE4; we created blueprint code such that 3D meshes can be loaded using either UE4’s stock procedural mesh methods or self-defined by using the free RuntimeMeshLoader and free RuntimeMeshComponent. For the ImageStimulus and MovieStimulus classes, we instead used the material property to determine their appearance. The range of materials that the user has at their disposal is almost endless, ranging from common textures to custom-made ones. We created custom materials via the UE4 GUI and added parameters to the materials that allow their properties to be changed. For example, we created an ImageStimulusMaterial for the ImageStimulus class which has a parameter “Image” expecting a UE4 2D texture. This texture can be created from an image or supplied by an image path (using Rama’s VictoryBPLibrary). The ImageStimulus class then sets this “Image” parameter from the StimulusSettings struct. We created similar custom materials to display videos for MovieStimulus class. For the GratingStimulus class, we used custom expressions within a custom material to create parameters (e.g., spatial frequency) that can be updated to change the appearance during runtime.

Other stimuli, e.g., moving stimuli or auditory stimuli, could be created by users with moderate Blueprint programming experience by inheriting from our base classes and adding new parameters. For example, in our experiments, we needed to display multiple stimuli at equal distances from each other and the Player. We therefore created a more specific stimulus class, MultipleStimulus. This class has parameters for the Player location, stimulus spacing, number of stimuli and their StimulusSettings. It then calculates the position of all the stimuli relative to the Player location and creates them there. It additionally records which of these stimuli is first reached by the Player.

### Control flow and states

**Fig 2:**
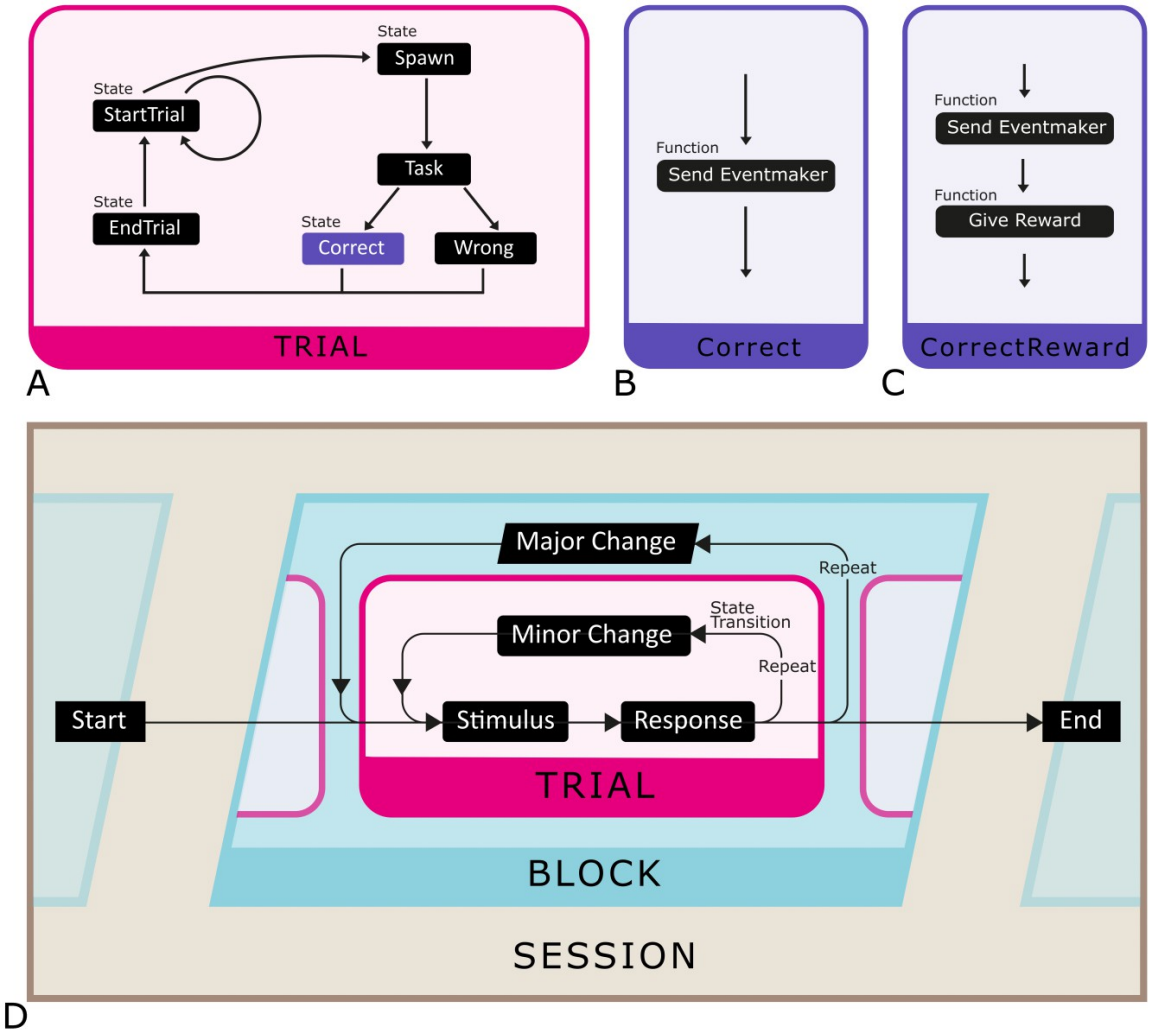
Control flow. (A) An illustration of a potential trial state machine. Arrows indicate the logic. Blueprint code for an example trial state machine is given in S2 Fig. (B) An example of a state “Correct” which sends an event marker among other functions (see Input/output). (C) An example of an alternative state CorrectResponse which has a new reward function added via drag-and-drop. (D) An illustration of the control flow of a DomeVR experimental task. A “Session” describes the loading of a Level for the subject to play. This loads a “Block” state machine containing a “Trial” state machine. Single states are shown as small boxes.

Immersive VR tasks often feature several decision points at which the subject’s interaction with the environment drives the next task event. A typical way to flexibly control the appearance of these task events as well as the flow between them (e.g., “correct”, followed by “end trial”), is by using state machines (see Fig 2A). A state machine defines basic sequences of actions and events that happen within a trial (such as showing a stimulus). Sequences can be rerouted depending on outcomes of a previous state (e.g., the outcome of the “task” state decides whether to next initiate the “correct” or “wrong” state). By using the same states as building blocks for different sequences (i.e., state machines), they can easily be reused for multiple purposes as well as edited and swapped in and out. For example, Fig 2B shows the “Correct” state, which sends an event marker denoting that the correct choice was made. By adding a reward sending function to this state we can make a new “CorrectReward” state (see Fig 2C), which can then be added to a new trial state machine. This system grants complete flexibility for the experimenter to create any type of task without needing to recode the entire task each time.

State machines can be nested within each other to build more complex task flows. In DomeVR all tasks are defined using state machines. The core state machine defines the event sequences of one trial (see Fig 2A and 2D). This core trial state machine can then optionally be nested within a state machine that defines more global task changes (e.g., changes in overall reward or which stimuli set are displayed). This container state machine is referred to as a block (see Fig 2D). To ensure that all the important state sequences are logged, we used Logic Driver Pro 2.4.6 [20] to create DomeVR states and state machines that automatically log task events, state start and stop times (see Behavioral logging) and can interface with DomeVR hardware (e.g., sending event markers or taking in eye tracking information).

### Timing control

As UE4 is mainly designed for smooth gameplay in an entertainment context, it does not have the millisecond timing precision necessary for neuroscientific experiments built in. It instead prepares multiple frames simultaneously to display them on the screen as quickly as possible. This means that there is no consistent delay between the time that a frame’s preparation starts, and the time that it is displayed on screen. To account for these variable delays, we tracked the frame currently being prepared and the frame being displayed with highly accurate QueryPerformanceCounter (QPC) timestamps. By sending event markers at important events during a frame and logging the same accurate timestamp of each event marker, we were able to adjust the timings of events to align with when their frame was displayed. The frame timing comes from the graphics card and can therefore only be accessed when the game is compiled and displayed full-screen.

Additional timing delays come from specifics of the hardware and software used with UE4 and need to be adjusted for. Three things are necessary for DomeVR; 1) the frame rate (in our setup this is 59.952 Hz), 2) the number of frames needed for the rendering pipeline (for DomeVR this is two (see UE4 dome projection)), 3) the delay from the time of the graphics card frame display to the time it was shown on the screen (e.g., our projector delay was measured as 18 ms). These together form an additional fixed delay that is accounted for automatically in our Python analysis module (see Log analysis).

In order to check that the modified timings were correct, we used a photodiode attached to the screen to detect the onset of each frame (see Photodiode timing measurements). To this end, we created an object in the VR that changes brightness on each frame when a state machine is running, and attached it to the Player to ensure that it was always in the same location in the VR as the photodiode. A widget in the GUI (see Graphical user interface (GUI)) allows the user to move this brightness object to the desired position on the screen. This widget allows users to place a photodiode in any convenient location on their setup and use the object to verify the timing of their frame onsets. The object sends event markers at each brightness switch. The timing statistics are then saved via DomeVRLog (see section below).

### Behavioral logging

In order to reconstruct what happens during a DomeVR task, we created a customized logging system, which we call the DomeVRLog. This is defined in a C++ class and implemented by an object attached to our custom Blueprint classes (e.g., stimuli, see Stimuli). It is also available as a separate plugin that can be added to other UE4 projects https://github.com/zero-noise-lab/TimedLog.

Two plain text files are automatically created in a folder specified by DomeVR whenever a new Level is loaded. One contains all information (hereafter referred to as the “continuous log”) and one contains only some of this information for convenience (hereafter referred to as the “abbreviated log”). The header of both logs contains basic parameters about the experiment as a whole, including the level start time, subject and experiment name (see S3 Fig). Each line of the file after the header contains a minimum of four columns. First is a column with the UE4 game thread time of the logged event, second is a column with an “Object identifier” (which is a unique number that can be used to track the identity of all objects in the UE4 world), third is a column specifying the type of the line, and finally there are a flexible number of columns depending on the line type. For example, an “event marker” line type contains a fourth column of the event marker id and a fifth with an accurate timestamp, whereas an “input data” line type contains a fourth, fifth and sixth column which respectively contain the forward, sideways and rotation input from an external input device. The plain text format makes it possible for a human to directly read the log file and understand the data stored in it, even if the information used to create it is lost, making it less dependent on specific file formats and thereby future-proof. In order to efficiently parse these large log files, we created an open source Python module, which is explained in more detail in Log analysis.

The continuous log is intended to store a complete account of all events that happened during the experiment. For example, it contains stimulus parameters as well as the location and time point of the creation and destruction for every DomeVR stimulus shown during the session, and the entry and exit times of all DomeVR states (see Stimuli and S4 Fig). Additionally, on each frame information about the Player and all other objects in the VR, such as their Cartesian UE4 coordinates and rotation, are recorded by a logging object. Sent event markers are also automatically logged (see Input/output). Finally, there is also the possibility to log custom information by using a Blueprint function that will write any text into the log. The timing of the log is based on the time of the UE4 game thread. However, as discussed above, the actual display time is delayed relative to this due to the additional time taken to render the scene on screen (see Timing control). From the 3rd game frame onwards, graphic card timing information is logged to adjust the game time to the actual screen time (see Timing control). Users also have the option to create an abbreviated log with a setting for verbosity level. At the lowest verbosity level, only major events are logged, e.g., state sequences. At the highest verbosity level, it is identical to the continuous log. This abbreviated log can be used for more efficient data analysis when only a subset of parameters are required.

### Log analysis

In order to analyze the data stored in the DomeVR log, we created a parsing module “parse_domevrlog” using Python. Using states and state machines means that the outline of a trial can be completely flexible, so that different trials may not consist of the exact same elements and events. We therefore needed the DomeVR log to be parsable in a similarly flexible manner. Our Python module allow users to 1) choose which log information they want to parse and 2) decide if they need the adjusted display timings or not. To choose which log information to parse, users select from clearly named functions that retrieve a subset of information (for example, “parse_position” retrieves all positions of a particular object, see S5 Fig). We handled the large file size of our human-readable text format (see Behavioral logging) by making use of memory mapping to ensure that loading the log does not use up too much computer memory. To request automatic adjustment of all log timings to screen time, a simple switch (“convert” is set to either True or False) was performed (see Timing control). This switch is set by default to True so that the user receives the already adjusted timestamps, as we assume that irrespective of experimental setups, experimenters will want to analyze when objects were displayed on screen rather than generated in the Game Engine.

### Dome projection

As mentioned above, DomeVR can be used with a lot of experimental setups. Here we describe functionalities more specific for our setup, namely, projecting the VR into an immersive dome screen, using a trackball as input into DomeVR, and our GUI.

#### VR visualization

**Fig 3:**
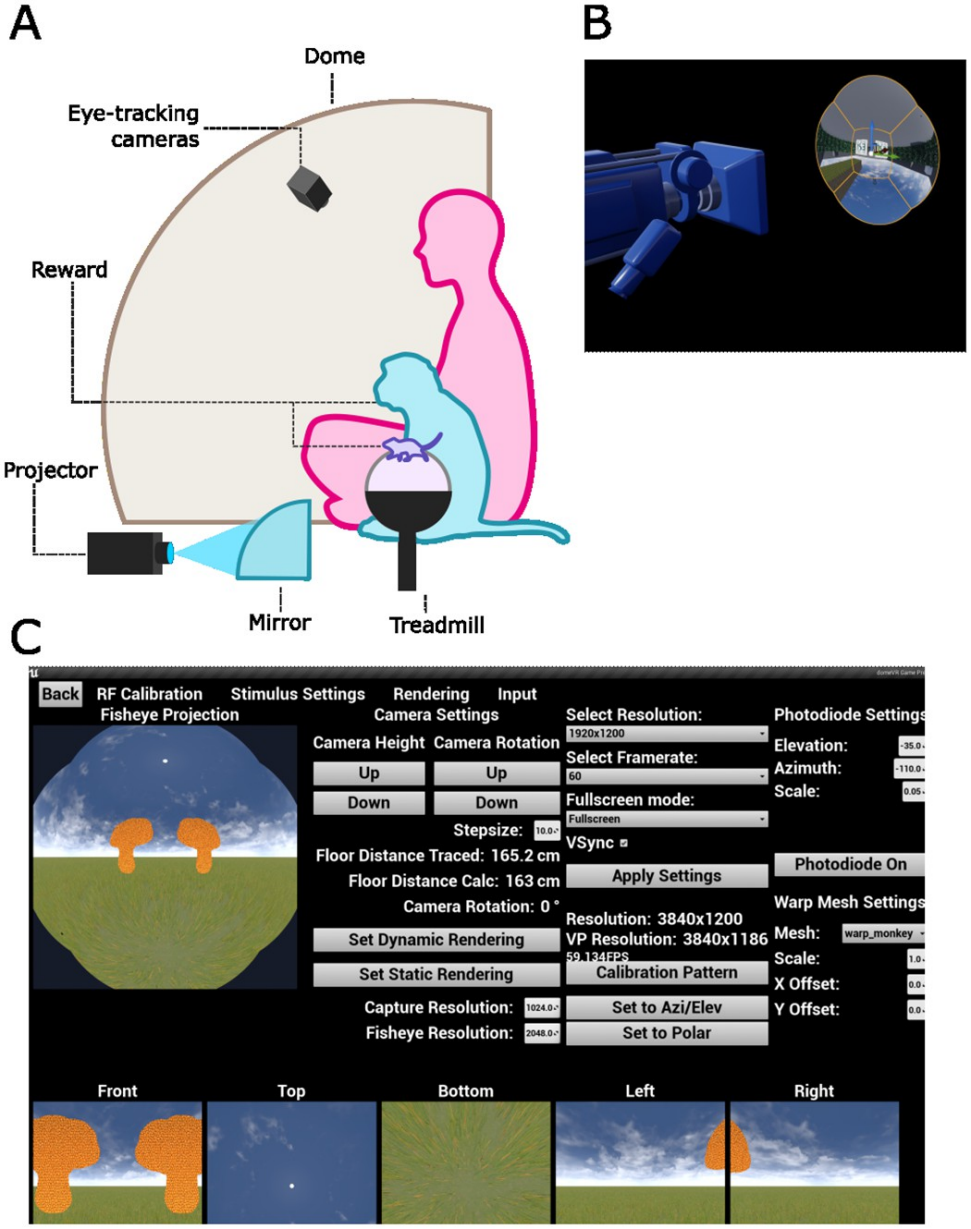
Dome projection. (A) Schematic of the dome setup. The projector is pointed at the curved mirror that projects the UE4 output onto the 250 degree dome. Input is received through a treadmill/trackball for mouse and primate subjects respectively. A camera placed within the dome can track behavioral input e.g., eye movements. (B) The fisheye view in UE4. The 6th scene capture component captures the warped fisheye view from the 5 different perspectives from 5 scene capture components. (C) The dynamic rendering pipeline UI allows the simultaneous viewing of the first 6 scene capture components and updating of their resolutions to find the best possible compromise between resolution and frame rate. Here demonstrated with a low resolution.

To create an immersive virtual reality (VR) environment suitable for a range of species from humans to rodents, in our experiments we project the VR onto a curved dome. This creates an immersive experience for subjects at the center of the dome by stimulating peripheral vision, even for species like rodents with lateral eye position and a correspondingly large field of view [21, 22]. We used a custom-made 60 cm radius spherical dome extending to 250° (fabricated by Fibresports, UK). The inner surface of the dome was illuminated using a 60Hz Canon XEED WUX450ST projector with a 1920x1200 resolution pointed at a 40 cm 180° acrylic (PMMA) convex mirror (see Fig 3, for details of this method see [23]).

#### UE4 dome projection

In order to project a VR environment onto a dome instead of a flat screen, the UE4 output needed to be warped to a spherical projection. While a dome projection method for Unreal Engine has not previously been created, we were able to base our approach on the method created by Paul Bourke for Unity [23], therefore it is described here only briefly. This method takes multiple camera views of the environment and warps them onto 3D objects called meshes to correctly distort them.

We attached a virtual five-camera rig to the Player that captured the scene from the five different perspectives needed to cover our 250 degree dome. These five views were applied to five meshes (downloaded from paulbourke.net) that distort the recorded images such that a single fisheye view was created (see Fig 3B) to be captured by a sixth camera. A final mesh (created using Meshmapper paulbourke.net) was needed to warp the fisheye view onto our specific mirror and dome combination (see Fig 3A). The fisheye view of the sixth camera was applied to this final mesh, and the resulting distortion captured by a final seventh camera and shown on the display. Since each view needs to be created before being applied to the mesh, this method created the two frame rendering delay mentioned in Timing control. This method is available as a separate plugin “DomeProjection” https://github.com/zero-noise-lab/DomeProjection, so that dome projection can be easily used in other UE4 projects.

We adapted the dome projection method to UE4 by placing the objects at a Level location far away from the Player (as opposed to the invisible layers described in [23]). Since the position of the final seventh camera did not correspond to the position of the Player, to enable procedural spawning of e.g., grass (which is dependent on the location of an active camera) and sound localization within the Level, an eighth virtual camera and audio listeners for sound were attached to the Player (see Unreal Engine 4). To easily reuse the dome projection in new Levels without having to recreate it, we loaded the objects for producing the dome projection as a sublevel within our other Levels. We additionally created a UI (see Fig 3C) to view the camera outputs and change the settings of the projection objects. For example, using this UI, the correct resolution for the scene capture components can be chosen to reach a desired frame rate and then saved to be used in future.

#### Dome coordinate logging

In order to calculate neural responses in visual areas, it is important to know exactly where stimuli are displayed. To achieve this, we needed to relate UE4 Cartesian coordinates to dome coordinates (a spherical coordinate system). We used a constant radial distance (the radius of our dome) and angles with position coordinate (0,0) at the front and center of our dome (i.e., the direction that the subject was looking). We created Blueprint code to convert between the angular dome coordinates and the UE4 Cartesian positions of our stimuli relative to the Player. We logged both sets of coordinates of our stimuli on each frame.

#### Receptive field mapping stimuli

DomeVR can also be used to do classical receptive field mapping. Our receptive field (RF) mapping stimuli are displayed relative to the dome coordinate system, and they must therefore be attached to the Player. The three main RF mapping stimuli were RFMapping, a strip of a sphere that was moved across the dome in a similar way as typical moving-bar mapping paradigms; RFMappingFlash, which produces small Gaussian blobs that can be flashed across different locations throughout the dome; and RFSparseNoise, which presents black and white squares of optional size and spacing at random locations across the dome. All three mapping paradigms have their own unique logging scripts to record the stimulus parameters, e.g., the center point of the RFmapping sphere strip on each frame. If subjects are centered in the dome and that their eyes are directed to dome coordinate (0,0), then dome coordinates can be directly equated to visual angles.

### Input/output

#### Ball input

To allow both human and non-human subjects to move through the virtual environment in a comparable and intuitive manner, we let all subjects control their motion in the VR by moving a trackball (by running on it in the case of rodents, and moving it with both hands in the case of primates). The movement of this trackball in X and Y was read out by two computer mice, which were then connected to the Unreal Engine via USB. For humans and monkeys, a GK75–1602B 75 mm trackball from NSI was used as input. Using a trackball as an interface with a computer is to our knowledge a novel method for macaques, but has been previously used by other non-human primates [24]. For the mice, we used a 20 cm diameter Styrofoam ball suspended in the air (modified method from [3]) with two Logitech G502 laser mice to read out its movement. A 3D printed holder for the ball was made according to open source schematics [25] (see Fig 3A).

Commonly used input devices (e.g., joystick, levers or keyboards) can be entered directly as movement input into UE4 (and therefore also into DomeVR) by adding them to the project input settings in the UE4 GUI. However, we needed to use an additional program for the multiple mouse readouts of our trackball to be interpreted as an input device without being interpreted as regular computer mouse input from the experimenter. To achieve this we used UCR, found on GitHub, with plugins interception and ViGEm to emulate an Xbox controller and block the corresponding computer mouse inputs from being seen by Windows and moving the cursor. The emulated Xbox controller was then selected as an input device in the UE4 GUI. Blueprint code was used to fine-tune gain changes between trackball readouts and VR locomotion etc. for the motion ranges of different subjects and subject species. The raw input values were saved to the DomeVR log for analysis. Rotational input via Turn Axis was deactivated for our experiments by removing the controller input for the Turn Axis in the UE4 GUI so that the Player would always face in the same direction. In experiments with different movement requirements, other input settings can be switched on and off, e.g., so that the Player does not move at all but only rotates on the spot. Since the movement of the trackball is continuous, we did not attempt to determine the exact timing of the mouse input relative to the raw input values logged in UE4. However, based on previous tests of UE4 we expect some small variable delay in the order of 6 ms [26], which we deemed negligible compared to the frame rate of our setup (60 Hz).

#### Event markers

We use event markers to distinguish particular events for synchronization with other hardware. National Instrument Data Acquisition cards are widely used in neuroscience to collect data with high precision. To facilitate the sending and receiving of precisely timed events and event markers, we built an Unreal plugin called UnrealDaqserverInterface to interface with NidaqServer. Via NI cards, the NidaqServer can send and receive these events via TTL pulses transmitted through very fast connections (up to 10 MHz). At the time of writing, NidaqServer supports eight NI cards with three types of connection (PCI, PCIe and USB); in our setup a PCIe-6321 card was used. This program was designed specifically with neuroscience research in mind and has commands to provide reward of different lengths (TTL pulses on a specific card channel) and send event marker codes that can be used to signal the precise timing of particular events in DomeVR (e.g., the start of a new trial). Each time an event marker is successfully sent, a precise timestamp is taken and logged (for more details, see Timing control).

#### Eye tracking

In order to create tasks that require the subject’s eye to remain within a particular area on the display (i.e., receptive field mapping), we built an Unreal plugin UnrealEyeserverInterface to interface with EyeServer, an open source program that connects to eye trackers. EyeServer currently supports iRecHS2 [27] and Eyelink (SR research), both of which are camera-based eye tracking systems that output the subject’s calibrated eye position at high frequency (500 to 3000 Hz) in real-time. Thus far, we have exclusively tested iRecHS2. Our plugin creates Blueprint functions which users can use to send requests to EyeServer. For example, they can request the current eye position, request that certain eye windows are tracked, and can query whether the eye is within the window. In addition, event markers signaling trial events can be sent to the eye tracker in order for the timing of the saved data to be synchronized with those same trial events in the DomeVR log. This will be done automatically if the user selects to load EyeServer. We also created a calibration task which displays points in angular dome coordinates for the subjects to direct their eyes to, so that the eye data output can be adjusted to the dome positions.

### Graphical user interface (GUI)

In a final step, we created a GUI to help scientists flexibly change their experimental settings, view the performance of the subject and debug issues with their experiment (see Fig 3C and S6 Fig). We created this GUI using the Unreal Motion Graphics UI Designer (UMG). Since UMG also uses Blueprints, changes to the GUI can be programmed easily with the same drag-and-drop principles. Currently, the GUI only works with a dome projection; however, adapting some menus of this GUI, e.g., the main menu and experiment control screen, to a standard flat-screen setup should be straightforward. The GUI is displayed on an “experimenter” screen, while the dome projection is displayed on the “subject” screen. Users can interact with the GUI while the subject is performing the task by clicking their mouse. In other stimulus presentation software only keyboard shortcuts can be used to change settings during stimulus presentation if one wants to maintain precise stimulus timing. To allow users to click on the experimenter screen for the interactive components, while simultaneously maintaining timing control of the subject screen, we needed to maintain full screen focus on the DomeVR window across two screens. To achieve this, we used NVIDIA Surround (NVIDIA Corporation) to bind the subject screen to the experimenter screen, creating a virtual single screen with a resolution of 3840x1200. The screen refresh time of both were synchronized.

### Main menu

The first part of the GUI, which starts automatically when DomeVR is run, is the main menu. This contains fundamental settings for starting a task, including entering the subject and experimenter name. Trial and block state machines and Levels stored in the appropriate folders are automatically detected and available to select from a drop-down list. As described in earlier sections, this GUI allows the user to set and save many specific settings for DomeVR. For example, on the first run for a new user, a pop-up requires the user to select photodiode position (see Timing control). Settings from the previous session are stored in the “Saved” folder of the project. When the RUN button is pressed, the selected trial or block and Level are run (as well as starting the external programs NidaqServer and EyeServer if requested) and the GUI switches to control screen.

#### Control screen

The control screen GUI is visible while the subject performs the task. It contains buttons for interacting with the running experiment as well as displaying information about the performance of the subject (see Performance charts and information about the current Level. The control screen also displays an interactive image of the subject screen, including the corresponding fisheye projection (see Fig 3C). Other interactive features include a Pause button, Variable Editor button (see Variable Editor) and a Settings button which brings up four tabs of other menus (see S6 Fig).

#### Variable editor

The variable editor displays all Blueprint variables from the running state machines and allows them to be changed while the task is running (see S6 Fig). This allows the experimenter to specify subject specific settings, e.g., difficulty level during training. For ease of use, a filter is applied by default such that only variable names starting with EDIT_ are displayed. The variable editor interface relies on a custom C++ class that reads out variable names and values, which are used to build up the variable lists in the GUI. When two state machines are active (e.g., a block calls a trial state machine) the variables from both can be accessed by selecting the corresponding state machine from the list of active state machines in the variable editor.

#### Performance charts

In the control screen, charts display the performance of the subject using the Kantan Charts plugin (found here). We created a bar chart, which displays the average performance per condition, and a line chart, which displays a sliding window of the performance per condition averaged across the last n trials (see S6 Fig). Performance was determined by calculating ratios of counts of different trial outcomes. What is counted can be any string, and is flexible and determined by the Blueprint code. We counted the number of times that a particular state was active (e.g., CorrectResponse) by using the name of the state as an outcome. This was made as simple as possible with Blueprint code that automatically counts the name of the current state. Even with little Blueprint experience, other methods of counting outcomes could be created. The outcome counts were not only used to create performance charts, but were also shown in the trial history on the right side of the ControlScreen GUI. This helps experimenters to keep track of task performance during training.

### Experimental regulations and conditions

We tested DomeVR on three species: humans, monkeys (*Macaca mulatta*), and Black6 mice (*Mus musculus*). All experimental procedures and housing conditions for the animals were in compliance with the German and European regulations for laboratory animal protection and welfare (EU Directive 2010/63/EU for animal experiments). The animal experiments were approved by Regierungspräsidium Darmstadt under the authorization number F149/2000. The human experiments were performed with permission from the ethics committee of the Medical Faculty of Goethe University (No:2021–252). Recruitment took place between January 11th and March 1st of 2022, and included obtaining written informed consent. The behavioural data described below are from one macaque, one mouse, and one human.

The macaque was pair-housed with a conspecific in a large cage containing an inside and an outside area. Environmental enrichment was provided daily in the form of branches, hanging ropes, large plastic cubes, feeding balls, etc, that were changed on a regular basis. The monkey was supplied with dry food every day and fresh vegetables and fruits on the days he was transported to the laboratory. The mouse was group-housed in an enriched environment containing mixed bedding, running wheels as well as a mix of cardboard and plastic igloos, and had free access to dry food and water. The group was kept in a reversed 12–12 day-night cycle (sunrise at 10 pm). Subsequent to surgery, the mouse was single housed, after full recovery from surgery he was given access to a social play cage with conspecifics 4–5 times a week for a minimum of 2 hours per week. Animal welfare was monitored by veterinarians, technicians and scientists throughout the study.

Animals were fitted with custom-milled headposts for the purpose of head fixation during this experiment. The headpost design and implant procedures for the macaque is documented in [28]. Briefly, a four-legged titanium baseplate was screwed into the skull under general anesthesia. After several weeks of osseo-integration, a titanium top part was screwed onto the baseplate in a simple procedure. During the experiment, the macaque was headfixed in a custom-made primate chair in a darkened booth. The headpost for the mouse is the subject of another manuscript [29]. Briefly, the animal was placed under isoflurane anaesthesia, shaved and given local analgesia on the top of the head. An incision was made and the skin on top of the cranium was removed, before the cranium was cleaned and the custom milled titanium head plate was attached using dental cement. General analgesia during and after surgery was ensured by adding Metamizole to the drinking water 5 days post-surgery.

The macaque in this study is currently still actively taking part in related experiments; the mouse was euthanised by anaesthesia with 5 percent isoflurane, followed by injecting an overdose (0.16 gr) of pentobarbital.

### Example experiments

We recorded the brightness changes on our dome using an amplified photodiode that was built in-house. We positioned the photodiode on the dome using a custom 3D printed holder. Both the photodiode signal and the 16 bit event markers were recorded at 30 kHz using an Open Ephys acquisition board. To record event markers at 16-bit resolution rather than the native 8-bit resolution of Open Ephys, we modified the Open Ephys system to accept 16 channels of TTL inputs to allow for a larger range of event markers to be recorded (https://github.com/zero-noise-lab/plugin-GUI). For the analysis in Photodiode timing measurements the recorded photodiode signal was low pass filtered to 500 Hz using a fourth order Butterworth filter. We tested DomeVR on using UE4.24 on custom built Windows 10 PCs from Alternate with 32 GB RAM, 1 TB SSD, AMD Ryzen 9 CPU and either a Geforce RTX 3090 or Nvidia Titan RTX GPU.

Our task built with DomeVR is the subject of another manuscript (in preparation) and is therefore described only briefly here. The human, monkey, and mouse were asked to distinguish two natural shapes, embedded in a grassy field, in a simple, two-alternative choice task. The shapes for the human and monkey varied smoothly between a starfish and a flower shape (see Fig 6A). On each trial, a blend between the starfish and flower was shown alongside the exact middle blend between these two shapes. The mouse had to distinguish between a jagged and a round leaf. The target stimulus (the shape more resembling the starfish and the jagged leaf respectively) was rewarded with a click sound for the human, a drop of juice for the monkey, and a drop of vanilla soy milk for the mouse. The distractor stimulus did not yield any reward and for the mouse, instead it was accompanied by white noise and a time-out. The human and monkey indicated their choice by moving to the stimulus with the 75 mm trackball; the mouse ran towards the stimulus on the floating Styrofoam ball (described in Input/output).

## Results

### Photodiode timing measurements

**Fig 4:**
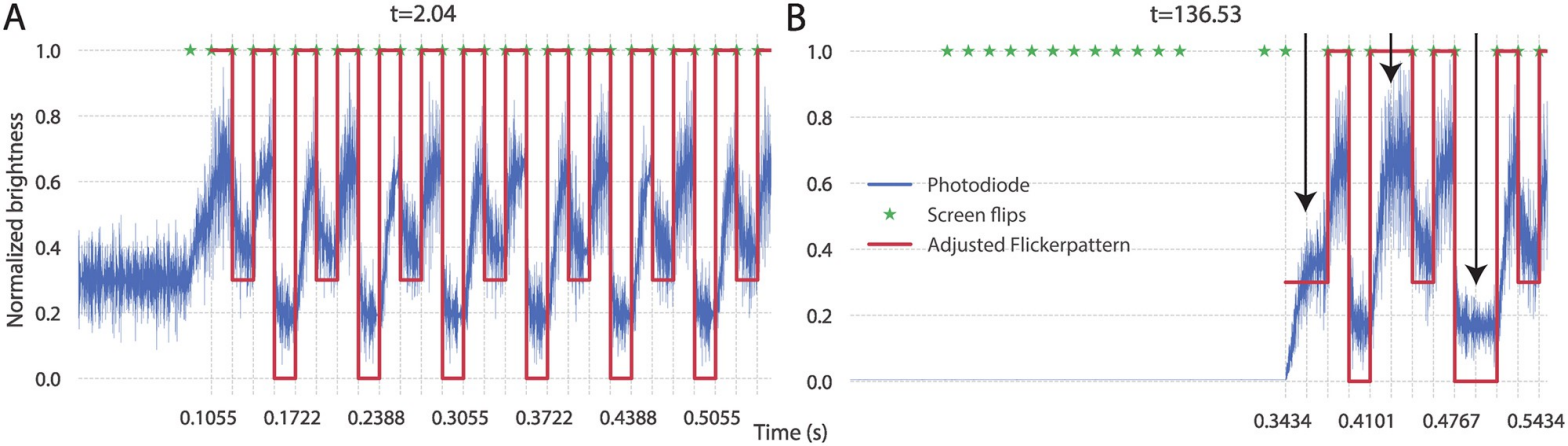
Photodiode results. (A) Photodiode recording of the brightness switching object with three brightness levels in the following pattern: 1,0.3,1,0. It initializes with a brightness of 0.5. Blue line is the recorded photodiode signal, green stars are all frame flips and red line is the software adjusted flickering pattern. (B) Photodiode recording of the flickering pattern shortly before and after the end of a pause, intended to induce frame skips (see arrows).

We performed photodiode recordings in order to verify the timing accuracy of DomeVR. In Fig 4 two short segments of such a recording are shown. As outlined in Timing control, a DomeVR brightness switching object (which changed its brightness on each frame) was positioned over the photodiode. We found that, as expected, the adjusted timing of the DomeVR brightness changes matched the recorded photodiode trace. This adjustment was performed by our parsing module (see Log analysis) according to the QPC timestamps from brightness switch event markers recorded in the log.

To ensure that our timing adjustments were correct even in the event of frame skips (stuck frames that are shown for twice the normal length of time), we caused them to happen by pausing and restarting the game. During a pause the whole screen was set to black. In Fig 4B three such frame skips can be seen indicated with arrows; the timings from the log follow them exactly.

**Fig 5:**
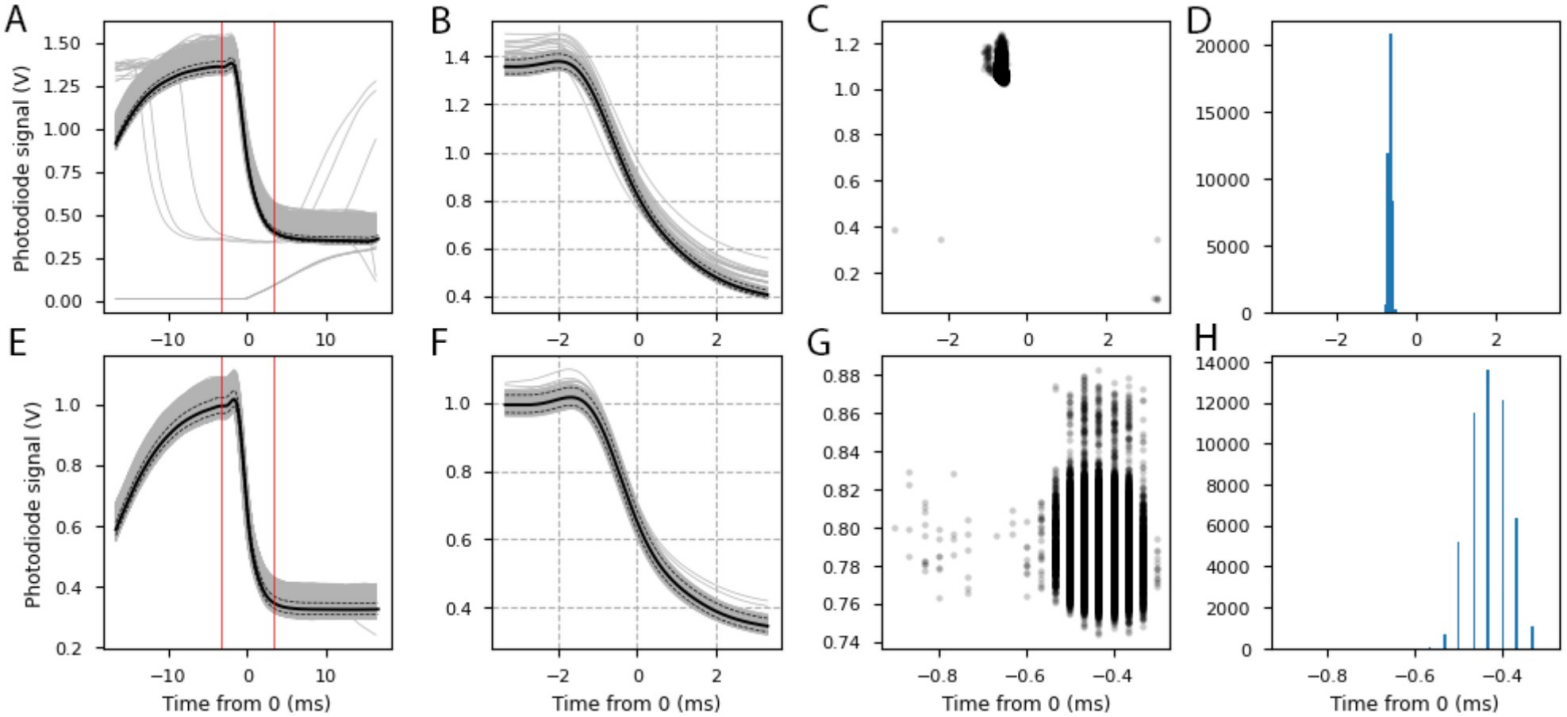
Photodiode traces aligned by DomeVR. (A) Analysis of photodiode alignment. Grey lines are individual alignments, black line is the median, dotted lines are 5th and 95th percentile. Red lines indicate analyzed time window. (B) As (A) but for an example 1% of the traces over the shorter analyzed time. Note the change in x and y axis. (C) Black dots are the maximum change in the photodiode signal of the analyzed area. (D) Histogram shows the counts of the maximum change times. (E-H) As (A-D) but the session was run on a second computer and DomeVR was not paused. Note the change in x axis in G and H. Here the maximum change varies by less than a millisecond and the limit of the 30 kHz recording resolution of 0.0333 ms is visible.

To measure the accuracy of the adjustment, we aligned the photodiode traces of the brightness change from 1 to 0 at the time indicated by the screen time alignment. In Fig 5A we see all traces in the session including frame skips (n = 41,904 transitions). To quantify the accuracy of this alignment, we measured the time of the maximum signal change. This resulted in a cluster of points around 0 ms with a photodiode signal of above 1 V (see Fig 5C). The median time of the timing distribution was -0.6667 ms, with the 5th and 95th percentile at -0.7333 ms and -0.5667 ms respectively. The fact that the frame flip appears to begin before time 0 suggests that our measured projector delay of 18 ms was in fact too long for these highly accurate alignments, and would ideally need to be measured to *μ*s accuracy. Therefore, even in this session of enforced frame skips, 90% of the traces were aligned to within 0.1667 ms.

For comparison, we repeated the analysis using a session on a second computer without pauses or frame skips. This time, all brightness transitions from 1 to 0 are well aligned (n = 50656, see Fig 5E–5H). Therefore, the time of maximum signal change is even more tightly clustered. The median was -0.4333 ms, with the 5th and 95th percentile at -0.5 ms and -0.3667 ms respectively. In this ideal session 90% of the traces were aligned to within 0.1333 ms and in both ideal and non-ideal sessions 98% of the traces were aligned to within 0.2 ms.

### Example case experiment

To demonstrate the utility of our setup to create tasks across species, here we show data from three example sessions across three species; human, macaque and mouse. We used similar Levels containing open grassy landscapes in all species, but the state machines are slightly different to comply with the needs of each species (e.g., reward and punishments do not apply for human subjects). The details of the task are outlined in Example experiments.

**Fig 6:**
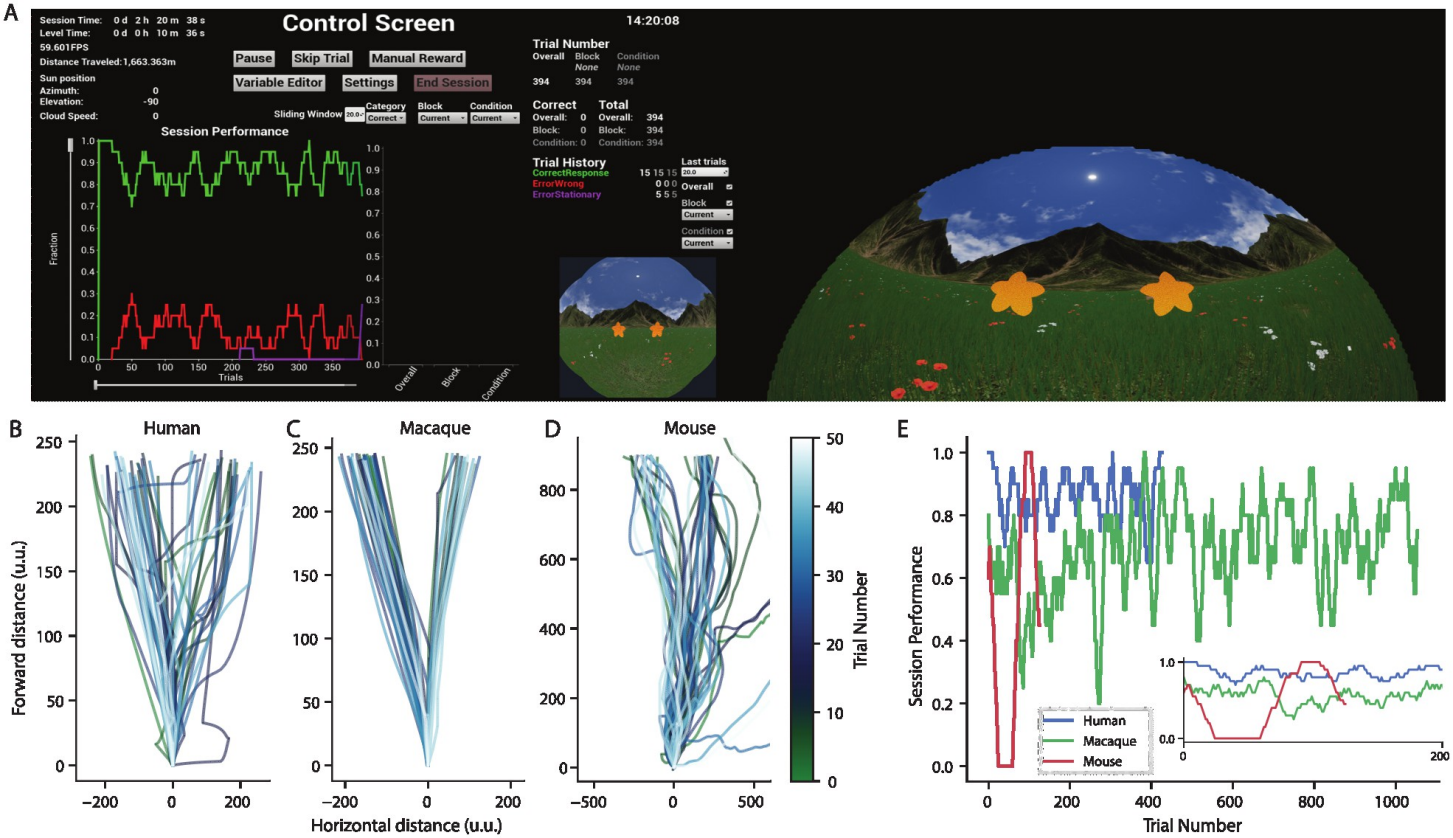
Example data from three species, human, macaque and mouse. (A) Dual screen screenshot of a human experiment (B) The path to the stimuli from the first 50 trials of a human. (C-D) As (B) but for the macaque and mouse experiments respectively. Different x and y axis in each are due to the different parameters of each experiment. (E) Comparative performance across trials for all three species. Performance is measured as the proportion of correct trials in a sliding window of 20 trials. For the human this is the same as the green line in (A). Inset shows the same performance measure for just the first 200 trials of each experiment.

In Fig 6A we see a screenshot from a human subject performing the task. On the left is the experimenter screen with ControlScreen GUI and fisheye view of the task during the session. On the right is the warped view projected onto the dome that the subject saw. In Fig 6B–6D the paths of the subjects for the first 50 trials in Unreal coordinates are shown. These paths demonstrate that all three species are able to accurately navigate in the virtual environment. Note the straight paths for the macaque, demonstrating that with many sessions of training, subjects are able to control a trackball with a high degree of precision. Finally, Fig 6E shows the performance at the conclusion of the human, monkey, and mouse sessions.

## Discussion

Here we present a versatile, easy-to-use toolbox for creating VR environments, making use of the powerful game engine Unreal Engine 4. UE4 is an effective open source game engine in use for easily generating photo-realistic VR environments, with drag-and-drop features for creating detailed scenes and built-in naturalistic lighting, physics, and more. We extended this to the flexible experimental control and high-precision timing needed for neuroscientific experiments. Our DomeVR toolbox has three crucial advantages. First, many components of it are modular and can therefore be used independently in other UE4 projects. Second, our toolbox can be used across several species by allowing different types of inputs, such as an air-suspended running ball for mice and eye tracking for monkeys, and by projecting the VR in a dome that covers the visual fields of the most commonly used model species. This is crucial to create a true, immersive VR experience in species with laterally positioned eyes, such as rodents. Third, our toolbox allows users with little to no programming experience to create experiments via the use of Blueprints. Blueprints are essentially a visual interface that transforms code into interconnected graphs, which represent typical game elements such as variables and events. They effectively give the user the speed and power of programming in C++, without ever being in contact with this conceptually difficult programming language. Other commonly used VR game engines, such as Unity and Panda3D, require proficiency in C# or Python respectively.

### Comparison with other VR toolboxes

Over the years, numerous toolboxes have been created to use VR in neuroscience experiments. Many of these have been developed specifically for investigating spatial navigation in humans (e.g., PyEPL [30], MazeSuite [31], PandaEPL [32], EVE [33], VREX [34], UXF [35], NavWell [36], bmITUX [37], Landmarks [38] and OpenMaze [39]). Almost without exception, these toolboxes use Unity to create their VR environments. On the other hand, VR experiments for mice are usually written directly in graphical programming languages such as Blender [6] and Bonsai [17], and typically feature simplistic, non-naturalistic VR environments. The only VR toolboxes for primates that we are aware of are the VR toolbox by [15]; and the Unified Suite for Experiments (USE; [16]), which is tested on primates, humans, and AI.

The VR toolbox by [15] is the only other VR toolbox for neuroscientists that is written with Unreal Engine. It runs on Unreal Engine 3, the previous version of UE4, which does not feature Blueprints but instead uses the visual scripting language Kismet. Kismet has a much reduced functionality compared to Blueprints. To make the toolbox as accessible as possible, it is controlled through text commands sent via TCP, which can be sent using any programming language. It therefore uses two computers, one for running Unreal and the other for controlling Unreal using these TCP text commands. This has the disadvantage that information sent via these predefined TCP text commands is limited compared to the many options available in the DomeVR GUI. The TCP connection also provides a hurdle for precise synchronization, which the authors overcome by synchronizing the computer clocks every 16 s through the Automachron software and logging the resulting time difference, which was 0 ± 3 ms. However, the accuracy of this synchronization was not tested for the timing of the events on the screen, which we synchronized offline in DomeVR. The toolbox supports any input device that can be configured as a mouse (e.g., a joystick or a trackball), and could therefore in principle be used for rodents also—though it has so far only been tested on a flat screen for humans and monkeys.

The other VR toolbox for non-human primates, the USE toolbox [16], is written in Unity. It offers excellent timing precision through the use of a piece of external hardware, the USE SyncBox. This SyncBox receives the timing of a photodiode and game engine frames in order to synchronize them offline. Our synchronization method is software based and therefore does not require a secondary piece of hardware, while still achieving sub millisecond precision. Apart from this, the USE toolbox is very comparable to ours in what it achieves, with our toolbox offering two important advantages: the projection into a dome, which makes it suitable for rodent research, and the use of Blueprints, which makes it possible for non-experienced programmers to nevertheless code experiments.

A promising toolbox for rodent VR research is BonVision [17]. BonVision is an open source software based on the Bonsai visual programming language, which can be used for displaying virtual reality (e.g., in a dome) as well as standard (2D) visual stimuli (e.g., on a flat screen). Its timing accuracy is in the order of 2 frames. The package contains several basic 3D objects such as spheres and cubes, and can import more complex natural scenes created in other VR programs such as Blender. However, it is unlikely to be able to achieve the photo-realistic immersive tasks we have created. Through its use of Bonsai, it is able to integrate with commonly used neuroscientific hardware natively and in a closed-loop manner. Similarly to Blueprints, it is suitable for non-programmers.

### DomeVR extensions

Owing to its flexibility, the DomeVR toolbox in its present form can already be used for many different experimental designs and in many different set-ups. For example, it is relatively easy to add features for spatial navigation tasks, such as mazes. This might be of interest to many labs, since VR is most commonly used in this domain. The first steps in this direction have already been taken; DomeVR includes large flat elements (in our case covered with a hedge material) that can be placed anywhere in the environment and combined to create a maze. Thanks to the use of Blueprints, it is also straightforward to add states that allow for a diverse task structure. Finally, the already built-in flexibility about the input devices into DomeVR (e.g., keyboard, lever, joystick, or trackball) enables its use across a diversity of experimental set-ups and model species.

In the future, the DomeVR toolbox could be further developed to make it even more generic. For example, the toolbox could be adapted for use on a flat screen or with VR glasses instead of a dome. The modularity of DomeVR and the plugins that we created will aid greatly in this endeavor. For instance, the ability to measure delays in the processing of input by UE4 would be useful for many labs. We have provided open source plugins for eye tracking, events and event marker sending, dome projection and logging with timing synchronization. Unfortunately, other parts of the toolbox had to be based on paid-for UE4 extensions, which are therefore not freely accessible but can be obtained for a relatively modest price if used non-commercially. We believe if more neuroscientists were to embrace Unreal Engine, the dual goals of photo-realism and ease of use for non-programmers could be met in an open source manner.

## Conclusion

In summary, we have created a VR toolbox that provides a more naturalistic environment than any of the rodent VR software around, and, due to its dome projection, is the only one that creates a truly immersive VR experience in primates. It features flexible experimental control and accomplishes extremely precise experimental timing. It allows non-programmers to code their task designs flexibly via the use of Blueprints, and many components of it are modular and can therefore be used in other UE4 projects. Although this toolbox was created primarily for use in monkeys and mice, it is versatile enough to be used in humans also, and in fact we have tested it on those three species, with very comparable results. We are excited to witness a general transition in systems neuroscience away from static task designs towards more naturalistic, active tasks, and hope that DomeVR will contribute to this transition by providing other neuroscientists with the means to easily implement such tasks.

## Supporting information

S1 Fig DomeVR classes. This UML diagram contains the relationship between most of the classes within the DomeVR toolbox.
(TIF)

S2 Fig Blueprint code of an example trial state machine. The grey entry box shows where the state machine begins and arrows connecting boxes show possible transitions between states.
(TIF)

S3 Fig Example log header. (TIF)

S4 Fig A two tick long excerpt of an example continuous log. (TIF)

S5 Fig Example usage of behavioral log parsing. (TIF)

S6 Fig Different tabs of the GUI. (A) The main menu has 3 necessary buttons to run the task: (1) Button to start running with the selected settings. (2) Drop down menu to find state machines within a folder. (3) Drop down menu to find Levels in the Level folder. (B-E) The various other tabs of the GUI that are present when a task is run. (F) The GUI for receptive field mapping parameters.
(TIF)
