# Supplementary figures and images for: DomeVR: Immersive virtual reality for primates and rodents
Source: PLoS One. 2025 Jan 16;20(1):e0308848. doi: 10.1371/journal.pone.0308848 (PMC11737658; doi:10.1371/journal.pone.0308848)

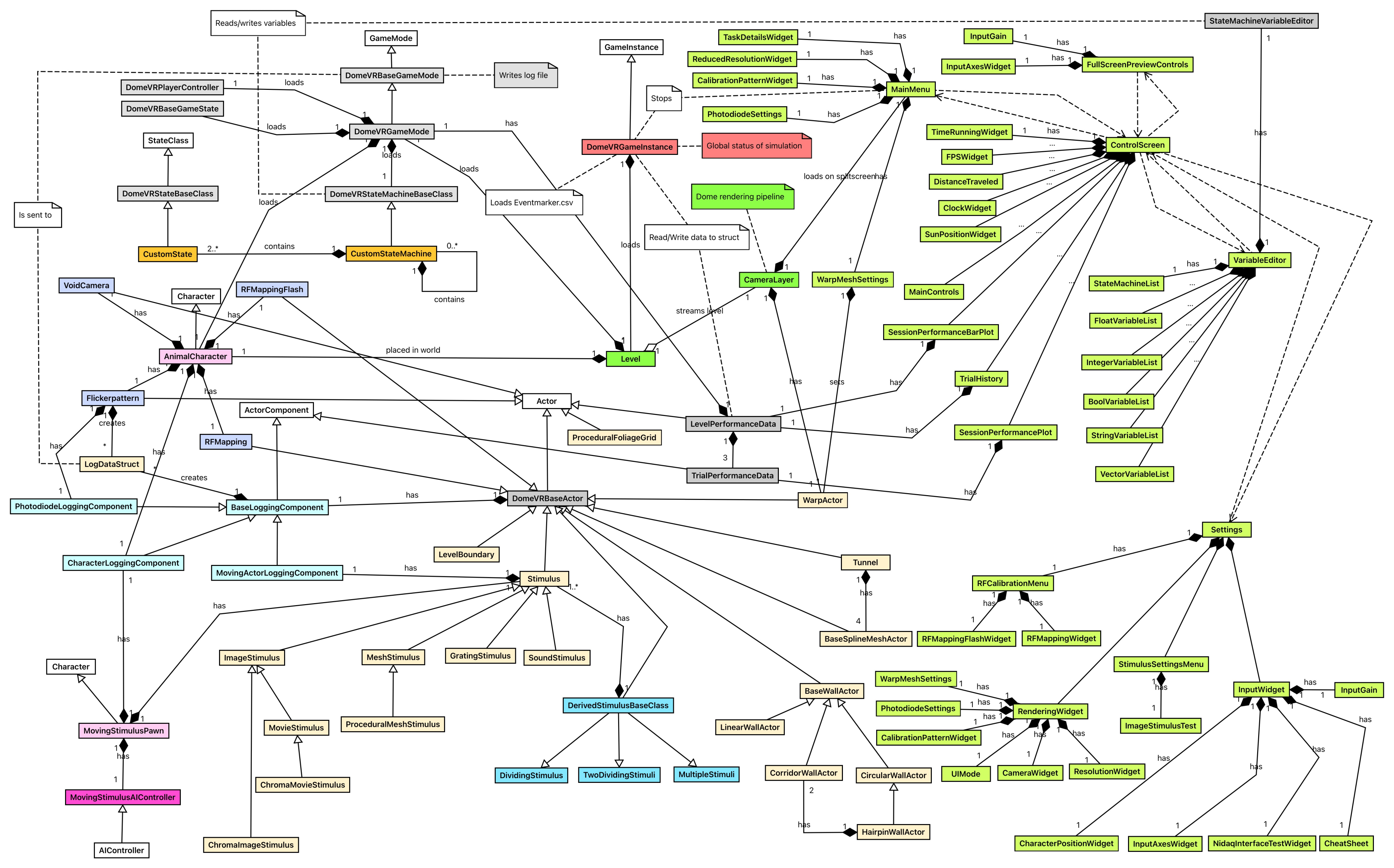

Supplement: S1 Fig — This UML diagram contains the relationship between most of the classes within the DomeVR toolbox. (TIF) [file pone.0308848.s001.tif]

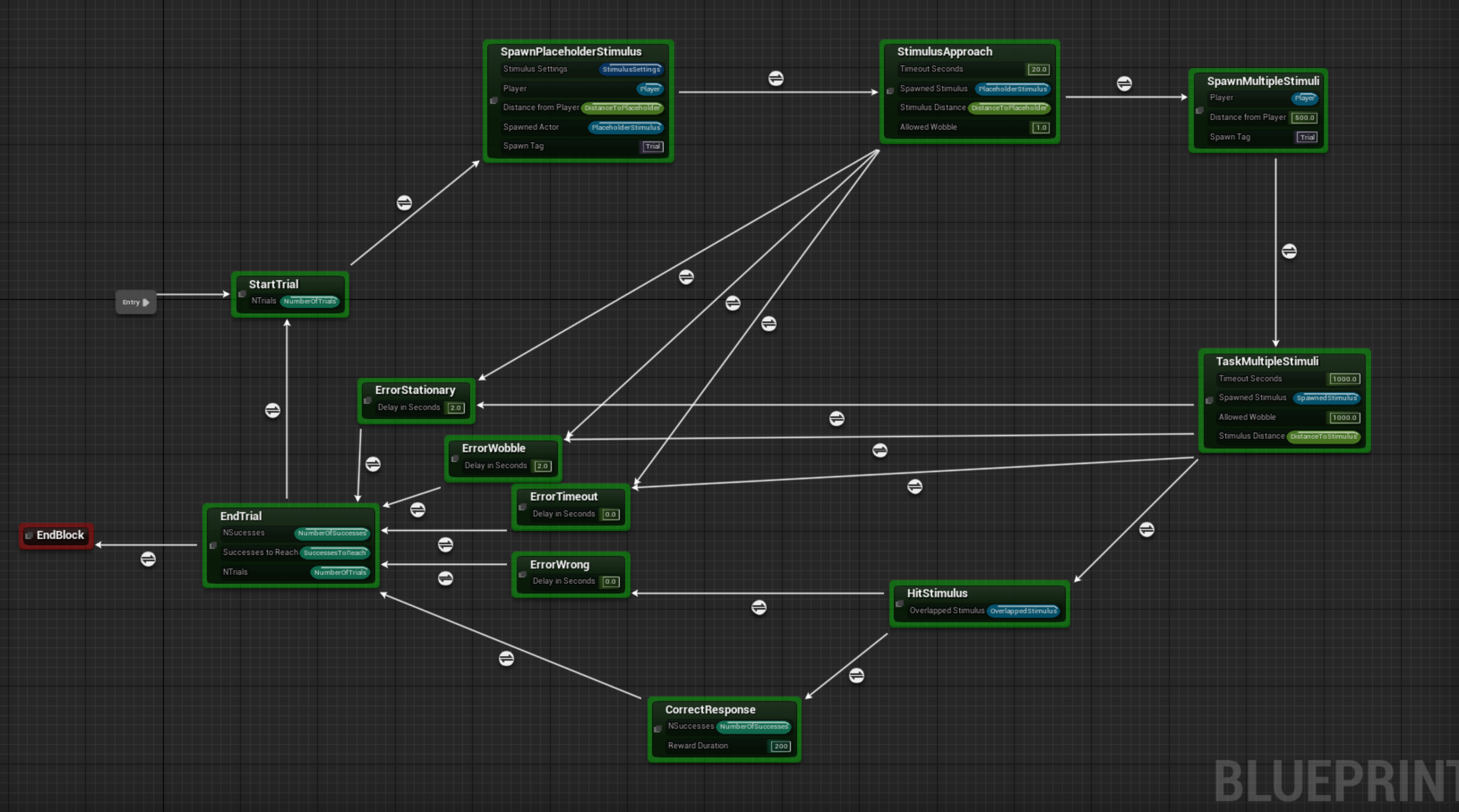

Supplement: S2 Fig — The grey entry box shows where the state machine begins and arrows connecting boxes show possible transitions between states. (TIF) [file pone.0308848.s002.tif]

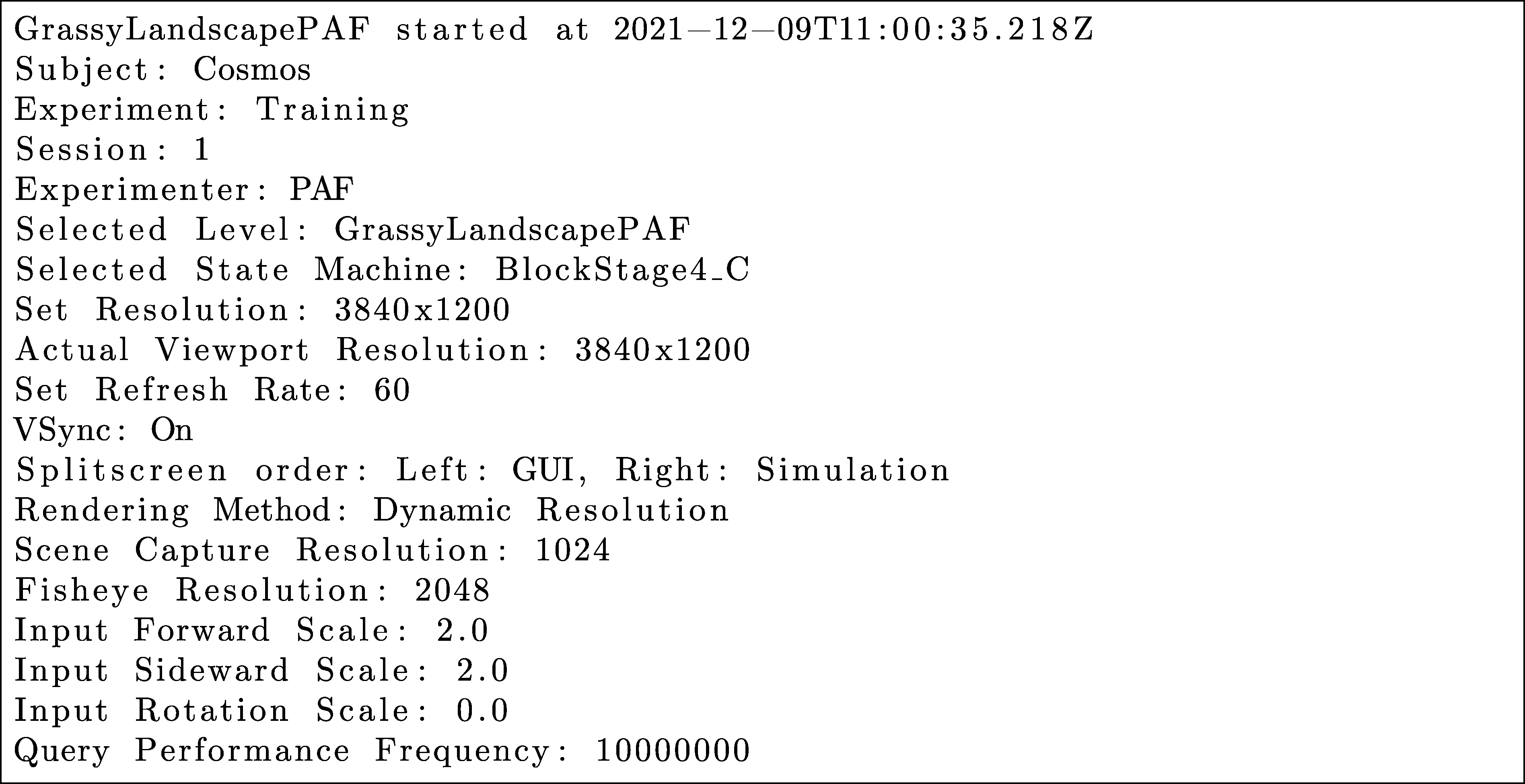

Supplement: S3 Fig — (TIF) [file pone.0308848.s003.tif]

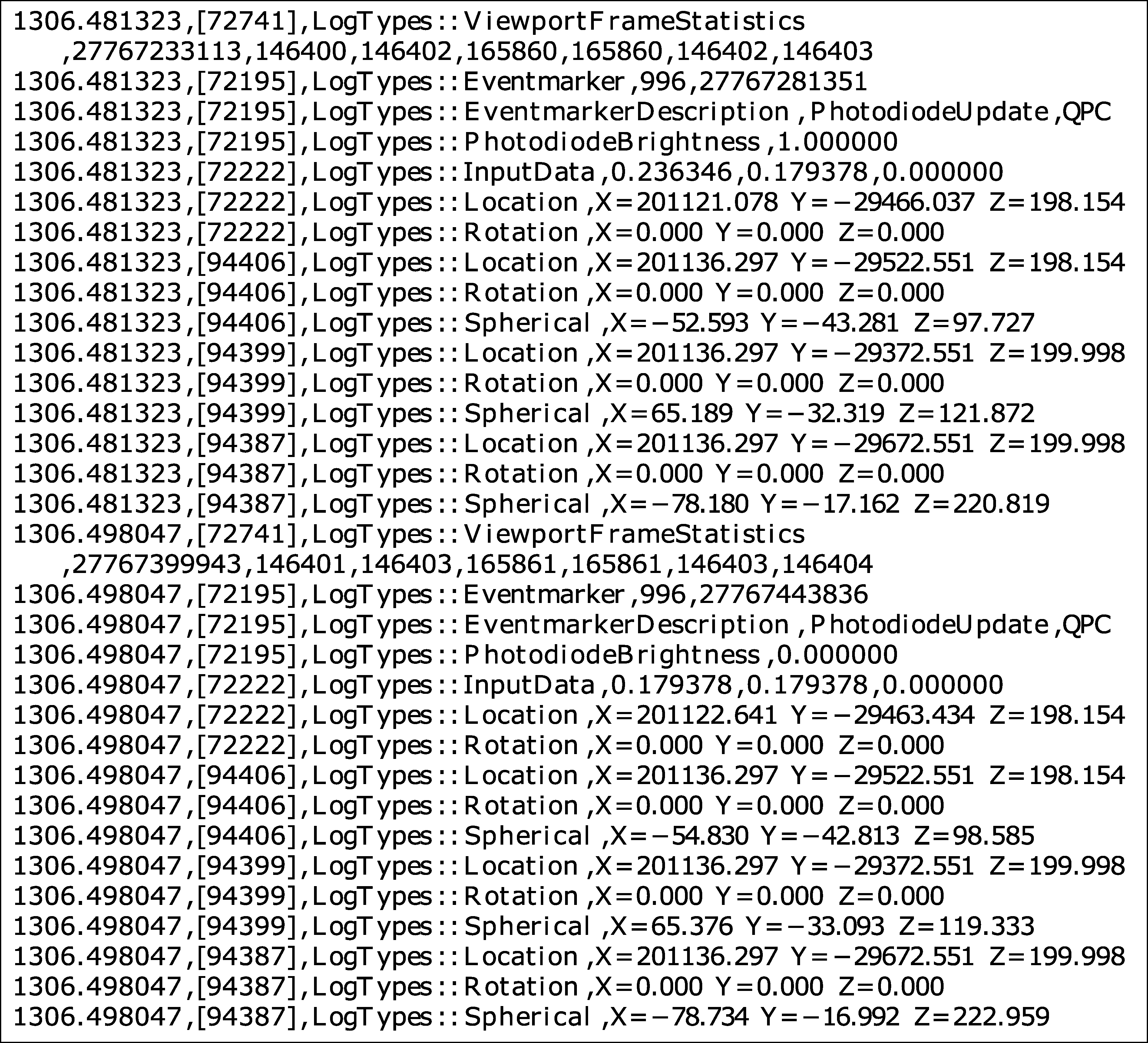

Supplement: S4 Fig — (TIF) [file pone.0308848.s004.tif]

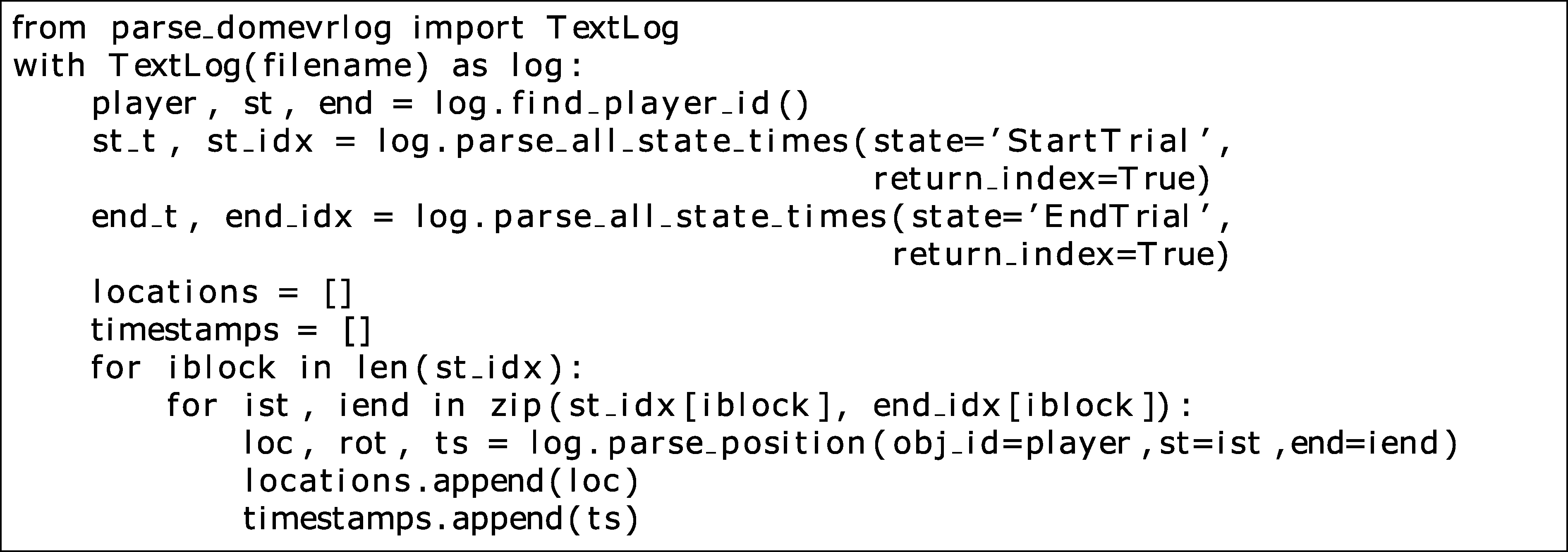

Supplement: S5 Fig — (TIF) [file pone.0308848.s005.tif]

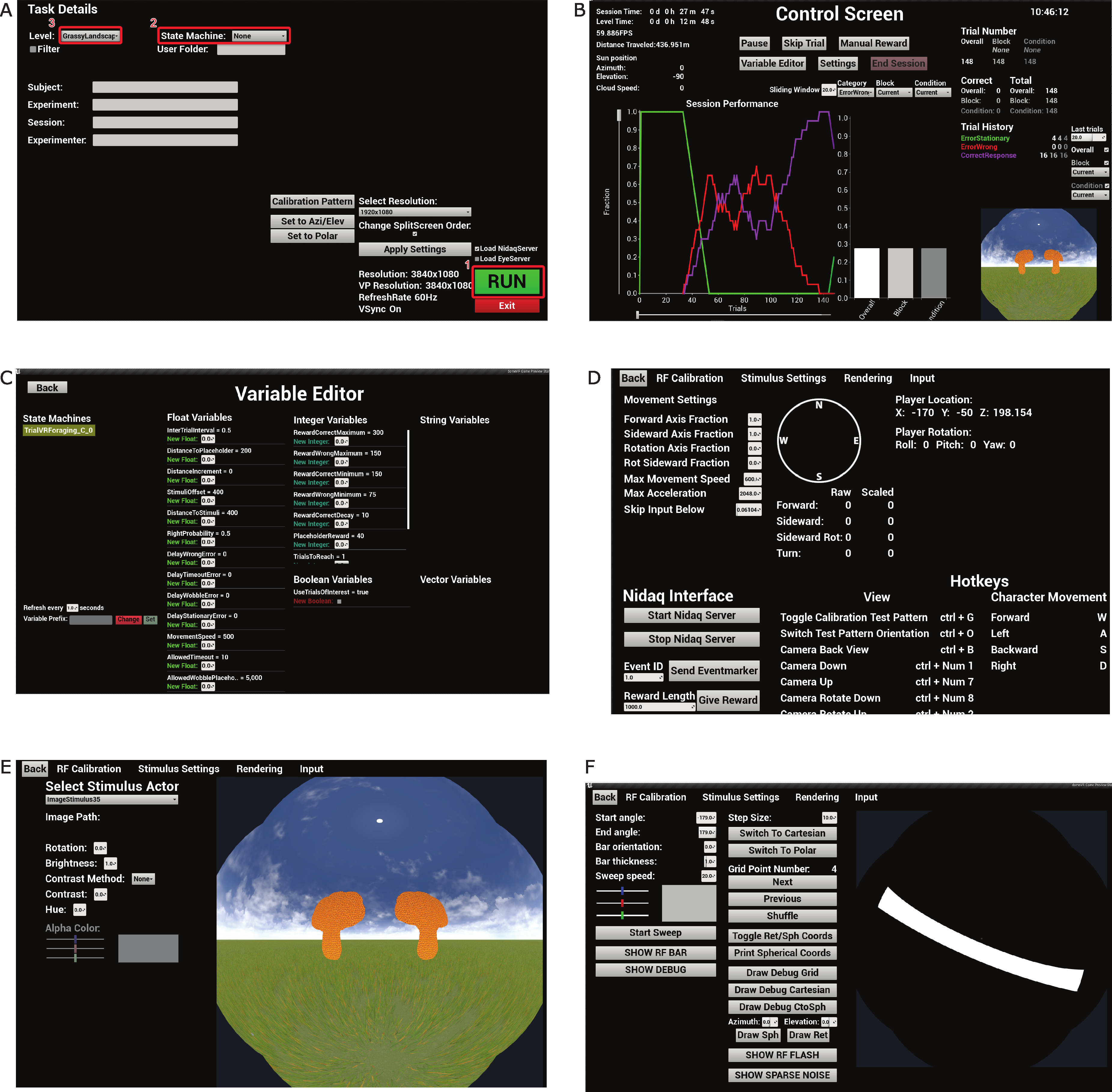

Supplement: S6 Fig — (A) The main menu has 3 necessary buttons to run the task: (1) Button to start running with the selected settings. (2) Drop down menu to find state machines within a folder. (3) Drop down menu to find Levels in the Level folder. (B-E) The various other tabs of the GUI that are present when a task is run. (F) The GUI for receptive field mapping parameters. (TIF) [file pone.0308848.s006.tif]
